# Parliament and the Profession

**Published:** 1919-07-12

**Authors:** 


### Parliament and the Profession.

### Pay of Army Nurses.

MR. RENDALL asked the Secretary of State for War why it was that, whilst all ranks in the Armies of Occupation have increased pay, the nursing sisters have received no increase whatever, although many have been abroad for years and some have recently been transferred from Salonika to Batum; whether he is aware that the sisters who asked to go to Batum have been compelled to pay their messing bills for the whole journey of 6s. a day, although many can ill afford it; and was he now prepared to treat the sisters and nurses working for the Armies of Occupation as fairly in the matter of pay as he has treated all other ranks. Mr. Forster replied that the Army of Occupation bonus is strictly limited to commissioned officers and enlisted men. Its extension to nursing staff would be a departure from this rule, but is under consideration. He would inquire into the question of the messing charges.

### Railway Fares of Pensioners' Friends.

COLONEL ARCHER-SHEE asked the Pensions Minister whether the near relatives of a discharged soldier undergoing treatment in a civil hospital for disabilities contracted on service are entitled to half-fare railway vouchers when visiting him; and, if so, to whom they should apply for these vouchers. Sir L. Worthington-Evans said that this could not be granted, but in the event of the condition of a patient becoming critical local committees would be justified in paying the cost of railway journeys undertaken by his nearest relative.

### Nurses Disabled in Former Wars.

THE question having arisen as to the inadequacy of pensions of those disabled in former wars, the Minister of Pensions, Sir L. Worthington Evans, announced that the Government had decided to increase pensions of officers and men so as to bring them generally to the level of the pensions given for disabilities sustained in the present war. This concession extends to officers, naval warrant officers, nurses, and men actually in receipt of disability pensions, and to revised rates arranged will be added the temporary bonus of 20 per cent. approved for cases arising from the present war under the same conditions. Concessions will have effect from April 1, 1919. The process of investigation must take some time, and the Minister asked for patience. Any pensioner, officer or nurse, who has a claim to increase can apply to the Ministry of Pensions (Officers' Award Branch), New North Street, London, W.C.

